# How you can help the JACMP in 2020

**DOI:** 10.1002/acm2.12839

**Published:** 2020-03-05

**Authors:** Michael D. Mills

In the January, 2020 issue, I discussed a retrospective of the JACMP on the occasion of its 20th anniversary. In this editorial, I would like to focus mainly on the past year, offer an assessment and ask for your help in further strengthening the JACMP. All of this is within the context that the JACMP is a core mission project of the AAPM, and that its publication partner, Wiley, continues to provide the highest level of professional services for this valued AAPM initiative.

In 2019, the JACMP published 264 peer‐reviewed articles, the most it has ever published in a year. The acceptance rate is about 47%, which compares favorably with other medical physics journals. The time from when the first potential Reviewer is contacted to the Associate Editor recommendation is a median 37 days; this is considered very efficient. The JACMP’s 2019 Impact Factor is 1.544, up from 1.301 in 2018, and 0.959 in 2012. The 2019 Impact Factor is for articles published in 2016 and 2017, then cited in 2017 and 2018; it was reported in late June, 2019. The Impact Factor reflects the yearly average number of citations that recent articles published in a given journal received. It is frequently used as a proxy for the relative importance of a journal within its field; journals with higher impact factors are often deemed to be more important than those with lower ones. Some argue that the Impact Factor for a clinical journal is of somewhat less importance than for science journals as cutting edge science is more immediate and newsworthy while the information contained in clinical journals is significant for a longer period of time, and may not be of substantial importance until significant time has passed after publication. Regardless, for the JACMP, higher Impact Factor numbers are good and represent increased penetration into the scholarly literature.

JACMP’s Associate Editors handle a median 9 articles per year. As most are aware, CAMPEP Continuing Education credits are available to Reviewers for reviewing an article, and to Associate Editors for managing an article through the review process. The article full text access by country is:

**Table:** 

Rank	Country	Percentage Access
1	US	33.9
2	India	7.4
3	UK	6.0
4	Australia	4.6
5	Japan	4.4
6	China	4.1
7	Canada	3.9
8	Germany	2.3

I list below JACMP’s 10 most downloaded articles as recorded within the Wiley system on November 1, 2019. This reflects the number of downloads for these articles since Wiley began publishing the JACMP on January 1, 2017:

**Table:** 

Article DOI	Article Title	Vol	Iss‐ue	Full Text PDF	Full Text HTML	Full Text Total
10.1002/acm2.12080	AAPM Medical Physics Practice Guideline 8.a.: Linear accelerator performance tests	18	4	2845	4062	6907
10.1120/jacmp.v16i5.5768	AAPM Medical Physics Practice Guideline 5.a.: Commissioning and QA of Treatment Planning Dose Calculations — Megavoltage Photon and Electron Beams	16	5	1931	3301	5232
10.1120/jacmp.v12i2.3368	Dose tolerance limits and dose volume histogram evaluation for stereotactic body radiotherapy	12	2	3663	1495	5158
10.1002/acm2.12146	AAPM‐RSS Medical Physics Practice Guideline 9.a. for SRS‐SBRT	18	5	1315	2777	4092
10.1120/jacmp.v12i2.3395	Computed tomography dose index and dose length product for cone‐beam CT: Monte Carlo simulations of a commercial system	12	2	318	2923	3241
10.1002/acm2.12469	AAPM medical physics practice guideline 10.a.: Scope of practice for clinical medical physics	19	6	821	2169	2990
10.1120/jacmp.v17i1.5799	Commissioning an Elekta Versa HD linear accelerator	17	1	995	1107	2102
10.1120/jacmp.v16i4.5412	CT protocol management: simplifying the process by using a master protocol concept	16	4	693	1048	1741
10.1120/jacmp.v15i1.4528	AAPM Medical Physics Practice Guideline 2.a: Commissioning and quality assurance of x‐ray‐based image‐guided radiotherapy systems	15	1	614	1014	1628
10.1120/jacmp.v16i4.5321	Determining the optimal dosimetric leaf gap setting for rounded leaf‐end multileaf collimator systems by simple test fields	16	4	1294	157	1451

Finally, the JACMP has an ongoing need to build its reviewer database. I ask all members of the JACMP community to take a few moments and help us by logging in to your profile to complete your areas of expertise. Many thanks for doing this. The instructions are below:

## 1 JACMP – Modify Areas of Expertise

Please follow these steps below to login, access the profile screen, and modify the profile screen.

1. Login ‐ ://jacmp.msubmit.net/cgi-bin/main.plex
  - If you have forgotten your Login Name, it is best to send an email to JACMPEditorial@wiley.com
  - If you have forgotten your password, click on “Forgot your password?” just above the Login and Reset buttons on the login page.

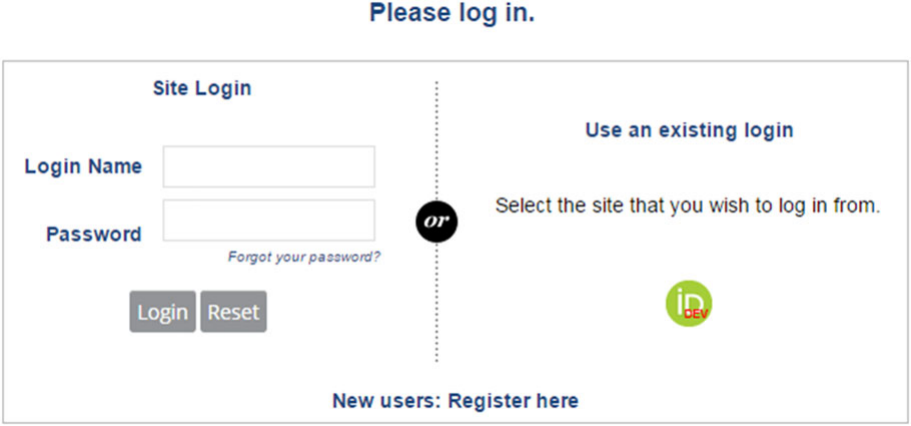

New users will be prompted to fill out affiliation/institution information as well as specifying areas of expertise before the registration process is completed.

- After logging in the site is separated into several subsections. In the bottom‐most subsection, entitled General Tasks, click on “Modify Profile/Password.”

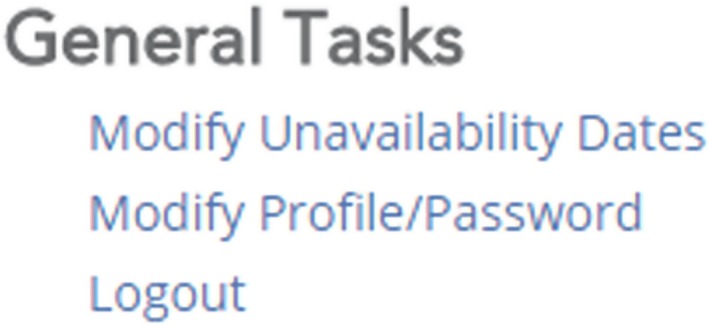

- This is the modify profile page. Here you are able to amend nearly every aspect of your profile and user settings. Scroll down to the bottom of the modify profile page to until you see “Will you consider being a Reviewer for this journal?”
  - Select by clicking on the circle to the left of your answer.

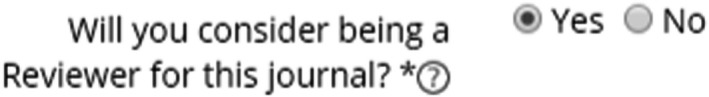

- After the reviewer question, you will see two sections for “Areas of Expertise.” The first section you can search by two methods.
  - Type key words into the “Search Areas of Expertise” text box.
  - Click on “I,” “M,” “T” to manually search the taxonomy.
    - When you find an area you wish to choose simply click on the text. It area you clicked on will be transferred to the box on the right (your selected areas).
- The second section is used for manually entering keywords you cannot find in the first section. Both sections are used by our database when Associate Editors search for potential reviewers.

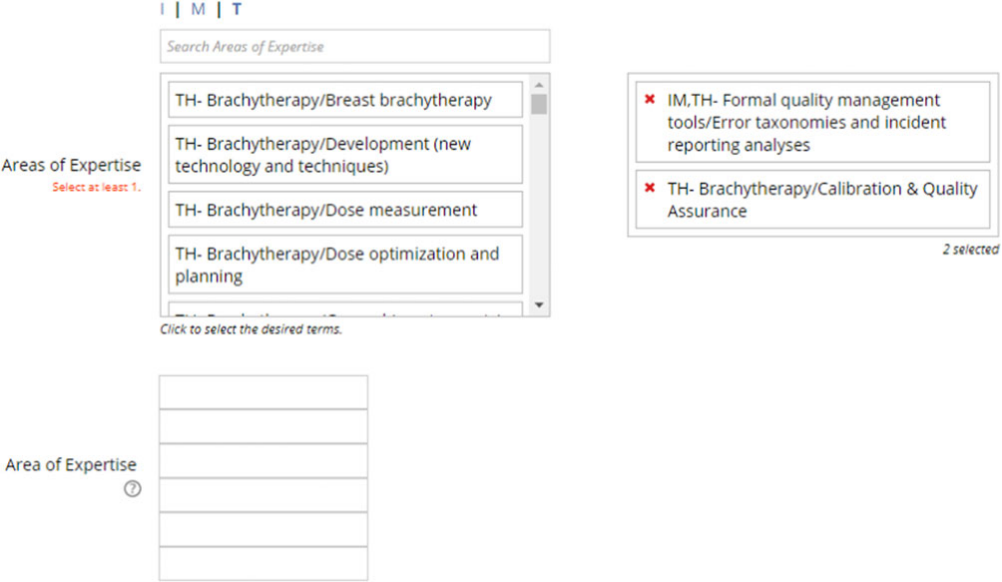

After the fields are modified click on “Modify Profile / Continue” at the bottom of the page to complete the amendments. This is also a good time to update your institutional information and link your existing ORCID profile. Any questions, concerns, or errors while modifying your profile should be sent to JACMPEditorial@wiley.com.

